# Bloom's syndrome in a 12-year-old Iranian girl

**DOI:** 10.4103/0971-6866.45003

**Published:** 2008

**Authors:** Naeimeh Tayebi, Hossain Khodaei

**Affiliations:** Genetic Research Center- Rehabilitation Comprehensive Center- Welfare Organization, Yazd, Iran

**Keywords:** Chromosomal instability, conjunctivitis, flat malar region, microcephaly, short stature, small mandible, thin and long face, telangectasia

## Abstract

**BACKGROUND:**

Bloom's syndrome, an autosomal recessive inherited disorder, belongs to the group of chromosomal breakage syndromes. The clinical diagnosis of BS is confirmed cytogenetically. Its frequency in the general population is unknown but it is common in eastern European Ashkenazi Jews.

**CASE REPORT:**

A 12-year-old girl was referred to us because of short stature. She was the second child of the first cousin marriage. She had a slender body frame, short stature, and microcephaly. Her face was long and narrow with prominent nose, and malar and mandibular hypoplasia. The spots of hyper and hypo pigmentation were observed in the trunk and limbs. Telangectasia spots were observed in some areas of the trunk. Additionally, generalized hirsutism was present in the whole body. Cytogenetic findings revealed an abnormality in the structural chromosome.

**CONCLUSION:**

This is the first BS case that has been reported in Iranian female population.

## Introduction

Bloom's syndrome (BS), also known as congenital telangiectatic erythema, was first described by Dr. David Bloom, a dermatologist, in 1954.[1]

BS is an autosomal recessive inherited disorder caused by mutation in the *BLM* gene that is located on chromosome 15 (gene locus, 15q26.1). Mutation in this gene causes errors in the copying process during DNA replication and results in a higher number of chromosomal breakages and rearrangements. This leads to the signs and symptoms of BS.[2]

The clinical features of BS are proportionate pre and postnatal growth retardation, dolichocephaly, facial sun sensitivity, telangiectatic erythema, patchy areas of hyper and hypopigmentation of skin, moderate to severe immunodeficiency manifested by recurrent respiratory tract and gastrointestinal infections, and increased susceptibility to wide range of cancers, especially to leukemia and lymphoma.[3]

BS is a very rare disease and its frequency in the general population is unknown, but it is common in eastern European Ashkenazi Jews with a prevalence rate of approximately 1 in 48000 persons. This is due to a founder effect, approximately 1% of the Ashkenazi Jewish population being heterozygous carriers for the *blmAsh* mutation. Also, it appears that men are affected by this syndrome slightly more than their counterparts.[4]

BS belongs to a group of chromosomal breakage syndromes whose clinical diagnosis is confirmed cytogenetically through demonstration of characteristic chromosome instability, but heterozygotes are not detectable by cytogenetic studies.[5]

There is no curative treatment for BS. However, a physician should carefully follow BS patients in order to ensure early diagnosis of cancer.[6]

In this paper, we have reported a new case of BS in an Iranian female which has been investigated at the Genetic department of Yazd Welfare organization.

## Case Report

**Figure 1:**
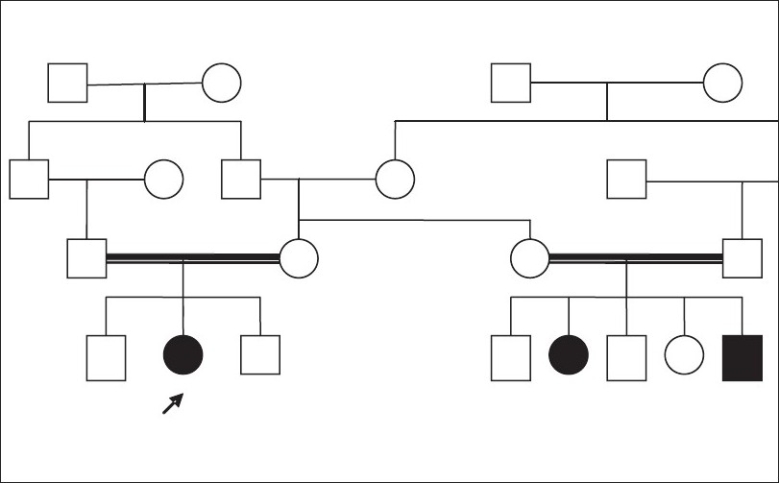
Family tree of the propositus

A 12-year-old girl was referred to Genetic Research Center of Welfare organization, Yazd, Iran, in June 2008, because of short stature. She was the second child of the first cousin marriage. All the other members of her family – parents, the first and third brothers – were healthy. At birth, the ages of her mother and the father were 23 and 28 years, respectively. There was no history of infections or medications during pregnancy. Family history revealed that two of her maternal cousins had similar signs and symptoms, though their features were comparatively worse [Figure 1].

She was born by vaginal delivery at 38 weeks of gestation with a low birth weight of 1700 g (<3^rd^ centile), height of 44 cm (< 3^rd^ centile), and a head circumference of 32 cm (10–50 percentile). She was discharged from hospital without any problem. Her developmental milestones were normal. At nineteen weeks of birth, for the first time, a urinary tract infection occurred and then she had to be repeatedly hospitalized for recurrent urinary, respiratory, and gastrointestinal infections.

**Figure 2:**
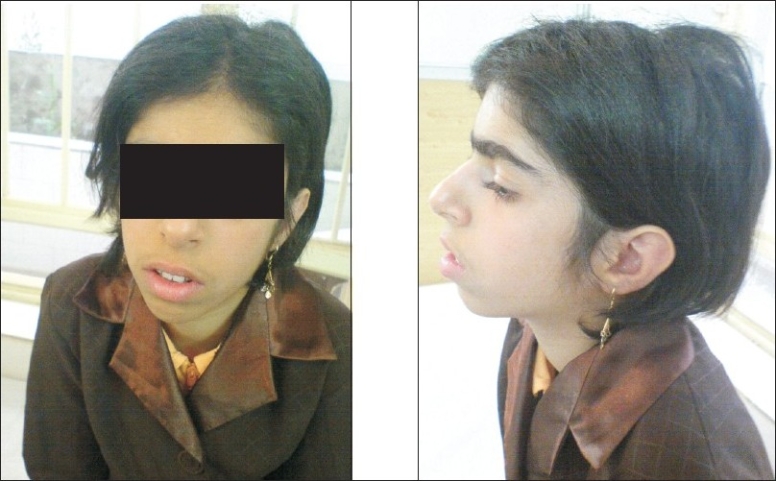
AP and lateral view illustrating the facial appearance of Bloom syndrome

Clinically, she was of slender frame weighing 25 kg (< 3^rd^centile), with a height of 133 cm (< 3^rd^centile), and a head circumference of 49 cm (<3^rd^centile). She had a long narrow face with prominent nose, and malar, and mandibular hypoplasia [Figure 2]. There was sun-sensitive erythema affecting the butterfly area of the face. Also, there were spots of hyper and hypo pigmentations (café au lait spots) on the trunk and limbs. Telangectasia spots were observed in some areas of the trunk. Additionally, there was a generalized hirsutism throughout the body. Ophthalmic and otic examinations were normal, except that conjunctivitis was seen in both eyes. Also, the teeth were normal. The structure and growth of the hair was abnormal. Her hair was fragile and fine. No deformity was observed in her fingers, toes, and joints. The external genitalia were unremarkable. Her intelligence quotient was normal.

Her neurological, cardiac, and para clinical (brain CT scan) examinations were all unremarkable. Her bone age was two years deficient of her chronological age. Routine biochemical tests and abdominal ultrasound were normal.

GTG-banded analysis of over 50 metaphases was abnormal. The following structural chromosome abnormalities were seen in four of the observed cells: 46, XX; chrb(1) (q42); 46, XX; chrb(X) (q21); 46, XX; chrb(7) (p22); and 46, XX; chrb(9) (q13). The final diagnosis was: 46, XX (46)/ 46, XX; chrb(1)/46, XX;chrb(X)/ 46, XX;chrb(7)/ 46, XX;chrb(9).

**Figure 3:**
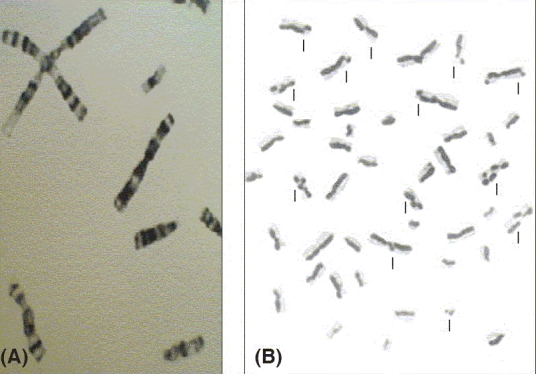
(A) G-banded metaphase chromosomes showing a four armed configuration. (B) SCE in bloom syndrome patient

Figure 3A shows a distinctive symmetrical quadriradial chromatid interchanges in one of the observed cell.

Also, sister chromatid exchange (SCE) analysis showed a mean of 80 SCE per cell in comparison to a mean of seven per cell for the normal control [Figure 3B].

## Discussion

BS is a rare human autosomal recessive disorder belonging to a group of chromosomal breakage syndromes. This syndrome is characterized by marked genetic instability, including a high level of SCE’s, associated with a greatly increased predisposition to a wide range of cancers commonly affecting the general population.

Kelly reported a case of BS in a black female.[7] In 1991, for the first time, BS was described in an Iranian Jewish male who subsequently developed myocardial disease.[8]

Szalay provided the first evidence of a genetic basis in BS. He described an isolated case in the child of first cousin parents and two affected siblings.[9] Then, German explained autosomal recessive inheritance of this syndrome.[3]

The patient, who is reported in this paper, is the second child of first cousin parents. Also, two of the children of her mother's sister had signs and symptoms similar to her. Her pedigree showed autosomal recessive inheritance in this disease.

She had a slender body frame, short stature, and microcephaly. Her face was long and narrow with prominent nose, and malar and mandibular hyperplasia. Also, sun-sensitive erythema was present on the butterfly area of face. In addition, the spots of hyper-and hypo pigmentation were seen in the trunk and limbs. In some parts of trunk, telangectasia spots were observed. Generalized hirsutism was present in the whole body.

Cefle *et al*, reported about an 18-year old girl diagnosed BS. Her ophthalmologic examination revealed mild lens opacities bilaterally,[10] whereas, in our patient, only conjunctivitis was seen in both eyes.

Mori *et al*, reported diabetes mellitus in BS,[11] while in our reported case the gGlucose level was normal without any signs of diabetic mellitus.

Clinical diagnosis of Bloom syndrome is confirmed cytogenetically by demonstrating characteristic chromosome instability.[5] The chromosomal study of this patient was 46, XX (46)/ 46, XX;chrb(1)/ 46, XX;chrb(X)/ 46, XX;chrb(7)/ 46, XX;chrb(9).
